# Growing Healthy Together: protocol for a randomized clinical trial using parent mentors for early childhood obesity intervention in a Latino community

**DOI:** 10.1186/s13063-019-3342-3

**Published:** 2019-04-25

**Authors:** Byron A. Foster, Kelsey Weinstein, Jackilen Shannon

**Affiliations:** 1 School of Medicine, Oregon Health and Science University, 3303 SW Bond Avenue CH16D, Portland, OR 97239 USA; 2 School of Public Health, Oregon Health and Science University and Portland State University, 3181 SW Sam Jackson Park Road GH230, Portland, OR 97239 USA

**Keywords:** Obesity, Preschool children, Latino, Low-income population, Mentors, Positive deviance, Randomized controlled trial, Behavioral intervention mapping

## Abstract

**Background:**

Latino children in the US experience high rates of obesity, increasing their risk of subsequent diabetes. There are few clinical trials among low-income, Latino families to test interventions that account for and address their unique situation.

**Methods/design:**

This trial, conducted in a Head Start (early childhood education) setting, randomly assigns children 2–5 years of age who have obesity by CDC (Centers for Disease Control and Prevention) guidelines (at least 95th percentile body mass index) and their parents to one of three conditions: (1) control, (2) parent mentor with an experimental curriculum, or (3) parent mentor with a standard curriculum (active control). We designed the experimental arm (2) using data from positive deviants: low-income, Latino families who had been successful in moving their child toward a healthy weight. Parent mentors are recruited and trained from the Head Start centers. Parent mentors then facilitate the teaching and coaching of parent–child dyads with weekly interactions over the course of a 6-month period. The primary outcome is change in adjusted body mass index z-score at the end of intervention and at 6 months post-intervention. Secondary outcomes include generalized self-efficacy, dietary intake, the home food environment, and reported physical activity.

**Discussion:**

This clinical trial contributes to the field by evaluating parent mentoring interventions that are potentially scalable for a population at high risk for continued obesity and subsequent morbidity and mortality.

**Trial registration:**

This trial was registered on October 31, 2017 (ClinicalTrials.gov identifier: NCT03330743).

**Electronic supplementary material:**

The online version of this article (10.1186/s13063-019-3342-3) contains supplementary material, which is available to authorized users.

## Background

Latino children have significantly higher obesity rates than the US population overall; recent national data show prevalences of 22% for Latino children and 15% for white children [1]. Children of migrant and seasonal farmworkers are at even higher risk given disparities in education and income [2, 3]. Early childhood obesity strongly predicts adolescent and young adult obesity [4, 5] and therefore confers a greater long-term risk for developing diabetes and cardiovascular disease [6, 7].

Effective interventions for early childhood obesity are limited, and primarily multidisciplinary, clinic-based models demonstrate effectiveness [8, 9]. Low-income, Latino families in particular face significant barriers to accessing health care [10]; therefore, the high requisite frequency of interactions for effective behavioral change for obesity [11] becomes a significant pragmatic feasibility challenge. Cultural perceptions and beliefs around weight among Latino families also present a significant challenge to acceptance of an intervention [12, 13], particularly for interventions delivered in a health-care environment [14].

Positive deviance is a complexity science-based approach that identifies individuals who are successful in a particular outcome despite being predicted to fail [15, 16]. Identifying the successful behaviors of positive deviants can inform an intervention for the larger population at risk. We previously identified core characteristics of positive deviants for early childhood obesity in a low-income, Latino population [17]. The potential benefit of using positive deviance is that by identifying strategies that already exist in the community, the intervention may be more scalable and overcome challenges related to cultural beliefs [12].

Parent mentors have been effectively used to encourage behavior change across a broad range of topics [18–20]. Parent mentors reduced asthma-related emergency room visits compared with controls [20], and parent mentors increased health insurance coverage and reduced unmet medical needs [18]. Parent mentors may be effective as they are able to leverage shared experience to identify motivations for behavior change and, with additional training, problem-solve effectively with families. Given the increasing evidence that obesity interventions based on a skills-based model are the most effective [8, 21], parent mentors were chosen as the delivery mechanism for this intervention. In this article, we describe the operationalization of findings from a positive deviance inquiry into a behavioral intervention delivered by parent mentors with planned evaluation of effectiveness via a randomized clinical trial.

## Methods/design

### Overall design and aims

This is a randomized controlled clinical trial testing different behavioral interventions to reduce adiposity among a low-income, Latino, preschool-age population. Parents of 2- to 5-year-old children enrolled in a child-care program for low-income families (Head Start) are recruited to participate. Parent–child dyads are randomly assigned to one of three conditions: control, parent mentor with an experimental curriculum, or parent mentor with a standard curriculum (active control).

### Setting, inclusion criteria, and recruitment

Head Start programs in the US serve low-income families and aim to promote school readiness via both individual learning environments and supporting the family’s health, educational, nutritional, and social needs. This particular Head Start program specifically works with populations of migrant and seasonal farmworkers in Oregon. The centers are located across the state in mixed peri-urban and rural areas where farming occurs, and over 4000 children are enrolled annually. Parents can enroll their children at multiple times during the year. As part of the usual care in this Head Start program, classes, offered at least monthly with child care and dinner provided, address parenting and child development, mental health, and nutrition, among other topics.

Inclusion criteria for parent–child dyads are parents of children who have obesity, defined as a body mass index (BMI) of at least 95th percentile using the Centers for Disease Control and Prevention (CDC) criteria, and are 2–5 years of age. Parents must be older than 21 years of age at the time of enrollment and may be of any weight. Both English- and Spanish-speaking families are eligible. Any child with a significant medical co-morbidity, including seizures, moderate to severe developmental delay, cerebral palsy, or taking medications for attention-deficit/hyperactivity disorder, is excluded given the potential for these to influence diet, physical activity, and growth. Current enrollment in a different obesity intervention program is also an exclusion criterion. Only one child per family may be enrolled in the trial. If more than one child per family presents for enrollment, one of the children is chosen at random for the enrollment and assessment.

#### Parent–child dyad recruitment

Eligible parent–child dyads are identified on the basis of the child’s BMI percentile documented by the Head Start community partner on intake into the program. Parents of eligible children are mailed an information letter with an opt-out postcard for them to return if they do not wish to receive any more contact about the study. If parents do not return the postcard, they are contacted and provided with additional study information via phone, text, or follow-up letter left at their respective Head Start center. If the parent expresses interest, we schedule a first visit where we measure their child’s height and weight and screen for co-morbidities. If both the child and the parent are eligible, the intervention is explained in greater detail, and parents are walked through an informed consent form in their preferred language (English or Spanish). Parents who consent to participate are asked to complete additional surveys (described below) and additional anthropometric measures are obtained on the child. Research staff consent, enroll, and randomly assign participants while blinded to the allocation sequence. Given the children’s age, they are not asked for assent.

#### Parent mentor recruitment and training

Potential parent mentors are identified within each early childhood care center by the Head Start staff as parents who have taken on an active or leadership role within the center. They are then recruited to participate on the basis of interest and availability. All parent mentors have a child attending the center and are required to have conversational-level English and Spanish proficiency. Parent mentors are assigned parent mentees after randomization with the expectation that parents mentor up to 14 parents per month.

All parent mentors undergo two-day training by the research staff. The first day of training covers general mentoring skills, such as active listening and supportive feedback, as well as confidentiality and the logistics of the program. The second day of training is specific to the curriculum they are going to use: either the positive deviance curriculum or the *We Can!* curriculum; each curriculum is described in detail in the following section. In regard to the positive deviance arm, the parent mentors themselves are not positive deviants per se; rather, they receive training in how to mentor other parents on the strategies used by positive deviants. Each potential mentor practices presenting portions of the curriculum. Parents participating in the mentor training are compensated for their time. Research staff observe parents during the training, and the final parent mentors are selected on the basis of the observations of research staff. Criteria used to identify candidates are bilingual fluency, engagement in the training, fidelity to the program content, and active listening skills.

### Intervention design

#### Experimental arm design

**Fig. 1:**
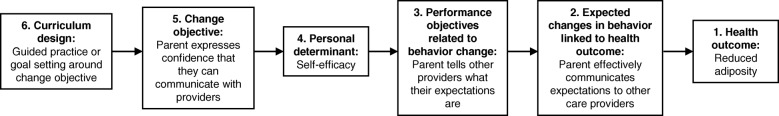
Flowchart of steps in intervention mapping adapted for this trial. Progress is shown from right to left with positive deviance inquiry informing the behaviors identified in step 2.

The experimental curriculum available in both English and Spanish was developed on the basis of findings from a positive deviance study that identified behaviors and practices from Latino families who were successful in changing their child’s weight status from obese toward a healthy weight [17]. After these behaviors were identified, we used a behavioral intervention mapping approach to create the curriculum for parent mentors to apply with parents in the experimental (positive deviance) arm [22, 23]. The four core behaviors that were identified and developed for this intervention were (1) parent creates a healthy home food environment, (2) parent effectively communicates expectations with other care providers, (3) parent supports small changes to increase children’s outside play time, and (4) parent is consistent about rules related to snacking and food. Intervention mapping attempts to explicitly tie theory and intervention program design through a series of steps that identifies what the behavioral outcomes are and the changes necessary to influence a given outcome [22]. (See Fig. 1 for adapted steps.) In this case, we had identified four core behaviors that we believed were tied to successful weight management in positive deviants; these were our behavioral outcomes (step 2 in Fig. 1).

Using the approach of intervention mapping, we identified the performance objectives a parent would need to meet in order to achieve the target behavioral outcome, and we broke down the behavior into micro-behaviors, similar to prior applications of intervention mapping (step 3 in Fig. 1) [22, 24]. As an example, the behavior of “parent effectively communicates expectations to other care providers” can be broken into at least six different micro-behaviors (performance objectives): (1) parent identifies priorities related to eating and physical activity, (2) parent expresses positive feelings about communicating expectations, (3) parent expresses confidence that they can identify and elicit goals, (4) parent identifies who providers are, (5) parent expects that providers will listen and try to meet expectations, and (6) parent expresses confidence that they can communicate with providers.

Next, we outlined the personal determinants (e.g., self-efficacy or outcome expectations) that would need to be modified to successfully facilitate the accomplishment of the given performance objective (step 4 in Fig. 1). We developed change objectives from a matrix linking the performance objectives to the determinants. For example, for the performance objective of the parent expresses confidence that they can identify and elicit goals, there are determinants of knowledge, attitude, self-efficacy, outcome expectations, and perceived norms (step 5 in Fig. 1). As an example of the matrix outcome, the change objective for this outcome under self-efficacy would be that the parent expresses confidence that they can communicate with providers.

Finally, we employed behavioral change theory and the best available evidence to guide an intervention affecting those health determinants driving the change objectives (step 6 in Fig. 1). Using the example of self-efficacy around provider communication in the preceding paragraph, we developed specific activities in the intervention to build self-efficacy among participants, such as guided practice, goal setting, and planning coping responses related to provider communication.

The intervention is delivered by using a model previously piloted and shown to be acceptable to Latino families with preschool-age children using parent mentors [19]. The parent mentors then follow up with each parent mentee over the phone, in person, or using text messaging on a weekly basis. This group also receives usual care in their Head Start setting.

#### Comparison arm design

The National Institutes of Health *We Can!* curriculum is used by parent mentors in the active comparison arm. Materials are available in both English and Spanish and this program has been shown to improve knowledge, attitudes, and reported behaviors among parents who have completed it [25, 26]. The curriculum was professionally translated into Spanish and proofed by a bilingual member of the research staff. This group also receives usual care in a Head Start setting.

#### Intervention frequency (intensity)

Parent–child dyads randomly assigned to the control arm receive health information from the Head Start center their child is enrolled in per the center’s usual care practice. Parent–child dyads randomly assigned to either parent mentor arm attend a 1-hour monthly meeting with their parent mentor and the other mentees in their mentoring group. (See Table 1 for a list of topics and activities.) The parents in the mentoring arms also receive individualized coaching on their goals for that month via weekly phone, text, or in-person follow-up with their parent mentor. The two intervention arms have 4 months of group meetings and individual mentoring followed by 2 months of individual mentoring on a weekly basis. The focus for the weekly communication is to encourage the identified behavior change and problem-solve through any potential obstacles faced.

**Table 1:** Topics covered in the two active intervention arms with the parent mentor–led curricula

Month	Positive deviance-based curriculum	*We Can!* curriculum
1	Creating a healthy home food environment	Overview, content, and structure of program. Motivation for eating well and moving more.
2	Effective communication around feeding and physical activity	Introducing the concept of energy balance
3	Supporting outside play time	Managing “energy in” of energy balance
4	Providing consistency, specifically around snacking	Managing “energy out” of energy balance
5	Individual goal setting, support, and troubleshooting (no community meeting)	Individual goal setting, support, and troubleshooting (no community meeting)
6	Individual goal setting, support, and troubleshooting (no community meeting)	Individual goal setting, support, and troubleshooting (no community meeting)

### Randomization

**Fig. 2:**
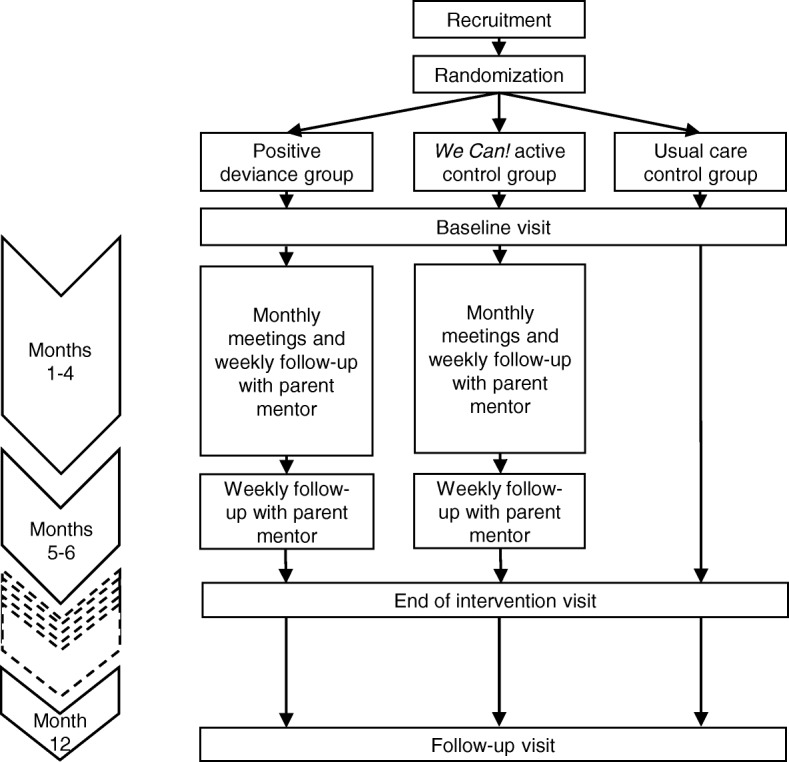
Randomization and flow of participants through trial over time

Parent–child dyads enrolled in the study are randomly assigned 1:1:1 to usual care, the experimental intervention, or the control intervention. (See Fig. 2 for steps involved in each arm.) REDCap (Research Electronic Data Capture) is used to facilitate randomization upon enrollment. REDCap also facilitates secure data entry and data quality assurance via data entry validation procedures. A block randomization scheme with block size varying from 6 to 12 was generated. Allocation concealment was maintained by having separate personnel review and upload the final allocation tables. Personnel randomly assigning participants are blinded to the actual group assignments derived from the allocation table. We do not blind participants by group assignment after randomization, and assessors at each follow-up are un-blinded.

### Sample size

Given the growth patterns of preschool-age children, weight maintenance has been recommended as the goal as this will lead to a reduction in adiposity with expected linear growth [27]. For the power calculations, we used a mean BMI z-score of 2.5 with a standard deviation of 0.7 for the baseline measurements, based on a previous trial in a similar popuation [19]. A BMI z-score of 2.5 is equivalent to a mean of 120% of the 95th percentile BMI for age and gender. Weight maintenance at this age leads to roughly a 0.5-unit reduction in BMI z-score over 4–6 months among children with obesity, a clinically meaningful reduction. We assumed a standard deviation of 1.0 for the 0.5-unit change in BMI z-score over 6 months. With these assumptions and to achieve 80% power at an alpha of 0.05, we would need 64 children per group to complete the 6-month assessment to detect this clinically meaningful difference of 0.5-unit reduction in BMI z-score between the two active intervention groups. We did not include a repeated measures correlation coefficient in our sample size calculation. We estimated a roughly 25% dropout rate by 6 months given the mobility of the targeted population, and so we aimed to recruit and enroll 80 dyads per group (240 parent–child dyads in total).

### Outcome measures

#### Primary outcome

**Fig. 3:**
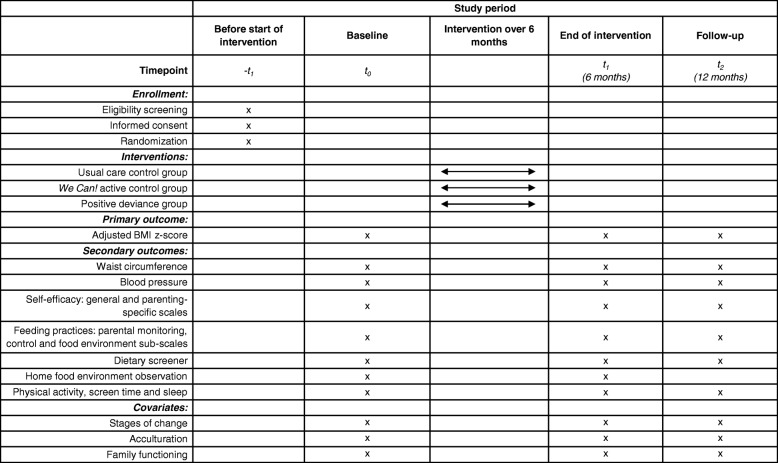
SPIRIT (Standard Protocol Items: Recommendations for Interventional Trials) figure: Primary and secondary outcomes, covariates, and assessment time points for parent–child dyads in the trial

The primary outcome is change in adjusted BMI z-score at the end of the active intervention period, 6 months post-enrollment. Adjusted BMI z-scores account for child age and gender and allow the evaluation of changes in BMI z-scores within the upper range of z-scores expected during this trial [28]. Adjusted BMI z-score is assessed in all three arms. The primary comparison is between the two active intervention groups at 6 months. The 12-month time point serves as an evaluation of maintenance of any changes post-intervention (Fig. 3).

We obtain the height and weight measurements for each enrolled child after removal of shoes and any outer garments (e.g., coat), allowing one layer of clothing to remain. Height measurements are obtained by using a stadiometer (model 233, Seca, Hamburg, Germany) and weight measurements with a portable, flat scale (model 869, Seca). Height and weight measurements are obtained in duplicate, and a third measurement is obtained if the two initial values differ by more than 1 cm for height or 0.5 kg for weight. The mean of the two closest measurements is used for BMI calculations, with the mean of all three values used if the measurements are equally-spaced.

#### Secondary outcomes

Blood pressure, waist circumference, self-efficacy, feeding practices, dietary intake, physical activity, sleep, and screen time are assessed by interviewer-administered survey at baseline, at end of the 6-month intervention period, and at the 12-month time point in all three intervention arms. The home food environment assessment is completed only in intervention and active control dyads at baseline and 4-month follow-up.

Blood pressure and waist circumference: Trained research personnel obtain blood pressure readings on child participants by using a standardized sphygmomanometer (model CONTEC08A, Contec Medical Systems, Qinhuangdao, China) on both arms in duplicate for each arm, as tolerated, and the mean of both values for each arm is used. Waist circumference is obtained by using the anterior superior iliac crest and umbilicus as anatomic landmarks, in triplicate with the mean of two measurements within 1 cm recorded or the mean of all three values if equally spaced.

Self-efficacy: The Generalized Self-Efficacy Scale (GSES) (12 items) [29, 30] is administered as well as parental self-efficacy questions related specifically to diet and physical activity practices and behaviors [31]. The GSES ranges from a score of 12 to 60 (higher scores indicate greater self-efficacy), and domains of initiative, persistence and effort are examined as subscales. The GSES has good internal consistency; Cronbach’s alpha values are 0.86 for the overall scale and 0.77–0.83 for the subscales [30]. The parental self-efficacy questions had been developed and tested previously among parents of preschool children, and the overall scale had a Cronbach’s alpha of 0.94 [31]. We added two questions on self-efficacy related to consistency in rules around snacking and communication with other providers; although we collected feedback from representative parents on these questions, these two queries have not otherwise been validated.

Comprehensive Feeding Practices Questionnaire (CFPQ) subscales: The intervention targets the food environment and potentially influences parental monitoring and control. Thus, we administer three subscales of the CFPQ to parents (environment, monitoring, and control subscales) [32]. The CFPQ is composed of a five-point, Likert-like scale ranging from “never” to “always” on responses related to practices and has a continuous outcome.

Dietary intake: The Block Kids Food Screener (BKFS), developed by NutritionQuest (Berkeley, CA, USA), is administered to parents to assess their child’s dietary intake. The BKFS is an abbreviated version of a food frequency questionnaire that queries 41-food items and can be used to compare consumption and patterns between groups [33].

Physical activity, screen time, and sleep: Outdoor physical activity is queried in a structured series of seven questions asking about days of the week spent engaged in outside play and then asking about specific times of the day with the reference period being the previous week [34]. Screen time is assessed by using questions from the National Survey of Early Childhood Health [35]. Sleep duration is assessed with questions about usual bedtime and wake times by using the method from the Zurich Longitudinal Studies to establish reference ranges for sleep duration in children [36]. These questions do not assess sleep latency or awakenings per se.

Home food environment: The home food and physical activity environment is assessed in the two parent mentor arms (positive deviance and active control) by using a standardized assessment tool adapted from a prior study [37]. This is a parent-reported observation tool that has been evaluated by comparing assessments of the home food and physical activity environment between observers, and only items with a kappa statistic of more than 0.61 are retained. Parent mentors administer the paper survey with parents in their homes and return it to the research office in a stamped envelope.

#### Other measures

The stages of change, acculturation scale, and family functioning scales are assessed by interviewer-administered survey at baseline, at the end of the 6-month intervention period, and at the 12-month time point in all three intervention arms. The qualitative interview is completed only with parent mentors.

##### Stages of change

Particularly in preschool-age children, parents underestimate their child’s weight or simply do not think it possible that their child could have obesity or be at an unhealthy weight [38, 39]. Therefore, a stages-of-change questionnaire that has been evaluated as compared with reported parental practice in diet and physical activity domains [40] is administered to parents to better understand how their participation in the trial and any changes in their child’s weight over time may be associated with differences in motivation.

##### Acculturation

The Brief Acculturation Scale for Hispanics (BASH) is administered to parents to assess their degree of acculturation [41]. The BASH has high internal consistency (Cronbach’s alpha = 0.94 overall). This measure assesses language use in different contexts. We added a question specific to dietary acculturation [42]. Parental country of birth and length of time in the US are also collected.

##### Family functioning

Given the targeting of family functioning in the intervention and the role that this may play in feeding practices, we administer the short version (six items) of the General Functioning Scale of the McMaster Family Assessment Device [43]. The short version that asks only about positive aspects of family functioning has been shown to have psychometric properties similar to those of the longer version [44]. The primary respondent, either the mother or father, completes this survey.

##### Qualitative data

We conduct individual, semi-structured interviews with all of the parent mentors at the end of their intervention period. The interviews probe their general experience, perceived effectiveness of the training, challenges and ways they overcame those challenges, and potential ways to modify the program.

### Data analysis

Given the relatively high potential for missing data because of attrition in the population enrolled over time, we plan a procedure of multivariate imputation by chained equations. Variables to be included in the multiple imputation model include BMI z-score of the child, child age, gender, family income, and language. This procedure operates under the assumption that the missing data are missing at random. A pattern mixture model will be applied to examine the informativeness of missing outcome data using time at 6 and 12 months to examine interactions with missing data patterns. The results of the pattern mixture model will be incorporated as a fixed effect in the linear mixed model (described below).

Using an intention-to-treat analysis, we will assess the primary outcome of mean change in adjusted BMI z-score at the end of the active period of intervention and use an alpha of 0.05. We will use linear mixed models to analyze the primary outcome of change in adjusted BMI z-score looking at the main effect of randomization group, time, and their interaction by using Wald tests for statistical significance and Akaike information criterion to assess model fit. A cohort variable, defined as the period of enrollment given multiple enrollment periods over the course of the study, will be included as a fixed effect. Baseline BMI z-score will be examined as a covariate. All models will be examined for the normality, homoscedasticity, and linearity of the residuals to ensure that the assumptions of the models are met.

Secondary outcomes will be analyzed by using similar methods, comparing by randomization group. We will adjust all secondary outcomes for multiple comparisons by using the Holm–Bonferroni method. We will track participation with a potential secondary analysis carried out stratifying by levels of participation. All quantitative analyses will be completed by using SPSS software version 24 (IBM Corporation, Armonk, NY, USA).

We will employ three regression equations to complete planned mediation analyses of intervention effects by the secondary outcomes. These include the main intervention effect on the primary outcome, change in adjusted BMI z-score, the main intervention effect on the mediating variables (secondary outcomes), and the combination of the intervention effect and mediating variables on the primary outcome. We will use a product-of-coefficients approach to examine the potential mediators [45].

Qualitative data will be analyzed by using software from Dedoose (Los Angeles, CA, USA). Coding and identification of emergent themes will be completed by multiple reviewers using a qualitative description approach [46].

### Ethics

Parents of participating children are compensated for their time in completing the surveys with $50 each for the baseline, end-of-intervention, and 12-month follow-up visits (6 months post-intervention). Parent mentors receive compensation at a rate of $50 per parent mentored per month. Given the low-risk nature, a data monitoring committee is not used. (See Additional file 1 for items addressed in this trial protocol.)

## Discussion

The primary goal of this study is to test the effectiveness of a behavioral intervention informed by a positive deviance approach for obesity reduction in young children. We used a positive deviance approach to identify key behaviors of successful families and then used behavioral intervention mapping to develop the experimental intervention. We employed this approach to identify health behaviors associated with successful weight management already in use by families of this demographic. Consistent with the usual approach of intervention mapping, we applied the method of breaking each behavior into a subset of performance objectives, then identifying health determinants of those performance objectives, and finally designing activities to address those determinants as change objectives. The behavioral intervention mapping approach has been used previously in obesity intervention development [24, 47]. To the best of our knowledge, using positive deviance and behavioral intervention mapping techniques together has not been done previously.

To best examine the effectiveness of the positive deviance approach to intervention development, we included an active comparison arm. This allows us to determine whether the content specifically derived from the positive deviance inquiry results in greater intervention effectiveness. The active comparison arm, *We Can!*, targets more traditional behaviors associated with a healthy diet, whereas the positive deviance curriculum focuses more on parenting skills (being consistent and effective communication).

There are a number of challenges to our approach. First, although our design will allow us to determine the differences in effectiveness of the positive deviance approach over both usual care and traditional comparison, it will result in smaller differences in change between some pairs of the groups, necessitating a larger sample size. However, in conjunction with our community partner, we anticipate being able to achieve our recruitment goal of 240 parent–child dyads. The potential benefit of including the *We Can!* group is that if there is no demonstrated difference between *We Can!* and the positive deviance group, it may be that simply having a parent mentor who provides social support for behavioral change and can reinforce topics is sufficient. The current study design is not a non-inferiority design, however, and cannot evaluate the equivalence of these curricula directly.

A challenge in the recruitment lies in the population served, namely that the migrant and seasonal farmworkers who make up a large proportion of the population move locations. Although the intervention can be adapted to the seasonal schedules and the parent mentors provide a viable and flexible method of contact, the challenge of retention remains. Maintaining contact to evaluate the 12-month time point, 6 months after the intervention has ended, may be a challenge.

Once this study is completed, the results will have implications for Head Start programs seeking to address obesity. One of the limitations in applying this design more broadly is the fact that parent mentors are provided with financial incentives. It will not be clear how feasible this study would be without the parent mentor or parent mentee subject payments for their time. However, the goal of this trial is to test effectiveness with fidelity relatively controlled and so we consider the financial payments necessary, particularly given the length of evaluations for the parent participant.

Most of the documented interventions to reduce obesity in the context of a Head Start program have been program-wide implementation of health promotion curricula [48–51]. This study represents one of the few targeted interventional studies in that it specifically identifies children with obesity for recruitment and participation. One might argue that most if not all parents could benefit from a parent mentoring program to support these parenting skills. However, given the resources required to support the parent mentors and the finding that (overall) many children do experience some reduction in adiposity with usual Head Start participation [52], we think this stratification is appropriate.

In summary, this article describes one of the few clinical trials designed for a low-income, Latino population of preschool-age children with obesity [49, 51, 53]. This demographic has one of the highest risks for continuing to have adult obesity and diabetes [4]. Data informing the best treatment approaches for this population are urgently needed and these types of clinical trials can help provide that information.

## Trial status

Protocol version 1 was posted on ClinicalTrials.gov on November 6, 2017, prior to any data collection. Recruitment began on January 1, 2018, and is expected to be completed on June 1, 2020; the completion for the primary outcome of BMI z-score at 6 months is anticipated on December 1, 2020.

## Additional file

Additional file 1: SPIRIT (Standard Protocol Items: Recommendations for Interventional Trials) 2013 checklist: Recommended items to address in a clinical trial protocol and related documents. (DOC 122 kb)

## Abbreviations

BASH: Brief Acculturation Scale for Hispanics
BKFS: Block Kids Food Screener
BMI: Body mass index
CFPQ: Comprehensive Feeding Practices Questionnaire
GSES: Generalized Self-Efficacy Scale
REDCap: Research Electronic Data Capture
